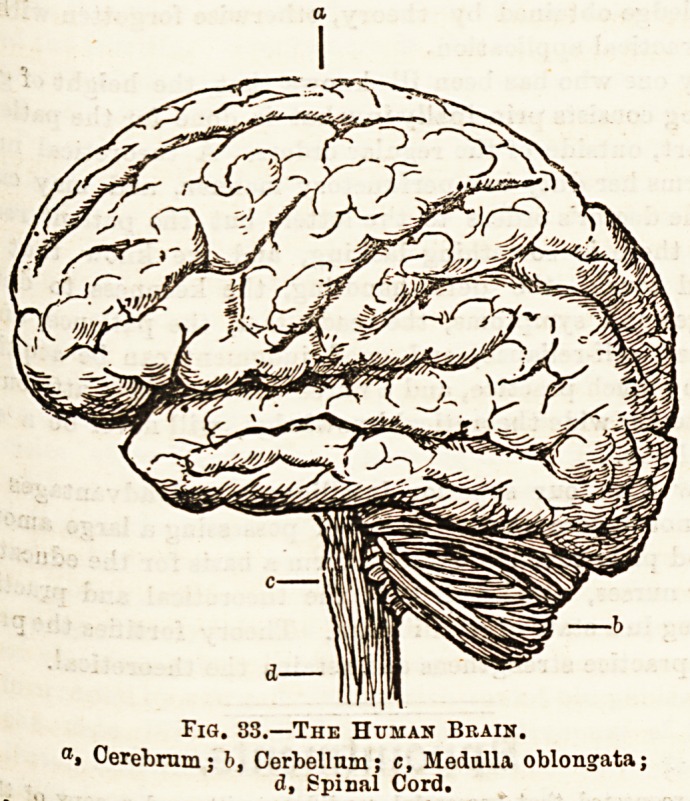# The Hospital Nursing Supplement

**Published:** 1895-07-06

**Authors:** 


### "The Hospital" Nursing Mirror.

Being the Extra Nursing Supplement of "The Hospital" Newspaper.

[Contributions for this Supplement should be addressed to the Editor, The Hospital, 428, Strand, London, W.C., and should have the word "Nursing" plainly written in left-hand top corner of the envelope.]

### News from the Nursing World.

### THE ROYAL VISIT TO ST. BARTHOLOMEW'S.

Excitement of a very pleasurable kind pervaded St. Bartholomew's Hospital on 28th ult., when their Royal Highnesses the Prince and Princess of Wales and the Crown Prince of Denmark visited some of the wards. The Royal party exhibited great interest in the patients individually, talking to a number of them in the course of their progress through some eight wards. "They spoke most kind to every woman in here," remarked a patient, "so feeling-like." "Yes, they've got sense to know sick folks like a friendly word," was the rejoinder. "But fancy that young-looking lady bein' a grandmother; it seems like a joke. You should just see *my* grannie!" Of course the wards as well as the patients had put on their brightest appearance, and the fine old hospital looked its best in the sunshine. One of the prettiest effects seen was in Elizabeth Ward, where the polished floors and furniture rivalled each other in spotlessness, the pale yellow flowers which formed the chief decorations being artistically arranged in glasses standing on deep red table cloths. As President of St. Bartholomew's Hospital, the interest taken by His Royal Highness the Prince of Wales in the institution is of long standing, and this visit, paid in company with "Our Princess" and her brother, the Crown Prince, was highly appreciated and will be long remembered.

### AT THE ROYAL FREE HOSPITAL.

The conversazione given on July 1st at the Royal Free Hospital attracted a large number of guests. The hosts were Sir Gainsford Bruce, chairman of the Committee, and Mr. Charles Burt, chairman of the Weekly Board, by whom the whole expenses were defrayed. The Imperial Orchestra and Mr. Foster's Amateur Glee Union performed a pleasing variety of music in the course of the evening. The exhibition of microscopes proved very popular, and the beautiful instruments on show in the lecture room appeared greatly to interest both students and nurses. Most of the honorary medical staff were present, as well as the members of committee and other friends of the hospital, including a number of well-known medical women and many of the present students. The evening proved such an undeniable success that, although the first, it will probably not be the last entertainment of the kind. Great credit is due to the secretary, Mr. Thies, for the admirable arrangements made for the 350 guests, in which he had the able cooperation of the matron, Miss Wedgwood.

### TRIALS OF TRAINING.

"When a ward is emptied of patients of course the nurses turn out too, as the place is dismantled and cleaned. We sleep where we can then. If there is not a spare bed, we perhaps have a sofa, a chair-bed, or make a companion uncomfortable by sharing her small mattress with her," explained a pupil-monthly nurse. "But surely you don't sleep in the ward at Queen Charlotte's?" asked her horrified friend; "the patients may be phthisical or otherwise unhealthy." "The authorities say it's for our own good to do so," the pupil replied wearily, "and they ought to know best; but a ward containing four mothers and four babies does not form an ideal dormitory for a tired day nurse. We pay fifteen guineas for a course of three months' instruction in monthly nursing, and we spend our nights, when we are on day duty, in bed in wards containing either two or four patients and their babies. We early learn the evil habit of sleeping through sounds of all sorts, as the night nurse is solely responsible for the patients. Of course the infants are seldom all asleep at the same time, which is trying to most of us." It is said that after these nights in the ward the pupils have to rise and dress themselves completely before going to see if they can secure the use of the one bath allotted to their service. Ward work from 6.30 to 7.30 follows, and afterwards breakfast is served, the supply of egg-cups and spoons being somewhat inadequate. It is to be hoped the committee will devote their attention to the evolution of a better system of training monthly nurses, and one which will include comfortable breakfasts for them before they go on duty in the wards, and will not teach them to disregard the cries of the babies or the restlessness of their mothers.

### NURSE OR SCHOOLMISTRESS?

The absolute authority which the medical officer is supposed to possess over matrons working under the Poor Law, is a sufficiently old-standing grievance to make a little evidence on the professional side of the question distinctly acceptable. The local press has furnished this in a report of the recent proceedings at Bromsgrove Workhouse. A nurse was taken from her duties in the infirmary to act as schoolmistress in the workhouse without the knowledge or consent of the doctor. His attention was drawn to the reduction of the nursing staff by finding the sole remaining nurse in charge of a child isolated by the matron for supposed measles, and going from it straight to the nursery. The case was presumably diagnosed only by the matron before being "isolated" by her in a room communicating with the general ward by large folding doors. The medical officer addressed a letter to the Local Government Board asking for their opinion as to the decision of the Bromsgrove Guardians "that the matron of the workhouse is to have independent control over the infirmary." One of the Guardians considered the doctor "was making a fuss about nothing," although the matron "had rather overstepped her duty with regard to her power to move the nurses." It will be interesting to the ratepayers to learn whether teaching sound children or attending to sick ones has hitherto been regarded as the primary duty of nurses in the service of the Bromsgrove Guardians.

### INEXCUSABLE SILENCE.

The lady who writes an article in the July part of the *Young Woman* on "How Not to Nurse," holds the views of Miss Nightingale, from whom she quotes, as regards the many qualities needful to make a perfect nurse. Unlike Miss Nightingale, however, this writer appears to possess only a limited knowledge of the modern trained nurse, and she certainly has little sense of her "duty to her neighbour." Otherwise how could she, as she asserts, go away from her old friend and leave him in the hands of the terrible woman who boasted to her that she was acting in direct opposition to the doctor's orders in giving her patient contraband food and stimulants; admitting visitors, and allowing the patient to get up "when the coast was clear." The gentleman eventually died, and the household "indignantly, and not without truth, accused her of having been the death of her patient." If this be a fact, both the writer of the article and the household are surely to blame for neglecting to tell the doctor the kind of woman to whom the dying man was entrusted. Stories such as these show the indifference of the general public to the remedy lying in their own hands. Neither doctors nor heads of private nursing staffs can fairly be blamed for continuing to employ nurses of whom patients and their friends are too indolent or cowardly to give truthful reports.

### UNPRACTICAL SUGGESTIONS.

In the course of the meeting of governors at which a new matron was lately appointed at Macclesfield Infirmary, a discussion of an extraordinary and, it is to be hoped, unusual character took place. According to the report in the *Macclesfield Courier and Herald*, the abolition of the office of matron was advocated by one of the governors. He thought a housekeeper might be appointed to look after the servants and domestic matters, while the house surgeon could see to the training of probationers and direct the duties of the nurses. Such an obviously impractical scheme would hardly deserve attention save for other remarks by the same speaker. These (reported as provoking laughter) show the management of the nursing department at Macclesfield to have been a task which the bravest of women might have shrunk from. After remarking that 34 men had held the post of house surgeon in the last 24 years, this governor asserted "that certain medical men in the past had been guilty of the impropriety of flirting with the nurses, and that consequences of a serious and scandalous character had in some cases followed; these were irregularities not entered on the minutes." That a higher salary might attract an older man is undoubtedly true, and a capable house governor or senior medical officer appears required at Macclesfield. Such an appointment would strengthen the position of the matron to an incalculable extent, but it would in no way obviate the need for having a fully-trained and experienced lady at the head of the nursing department.

### PREPARATION FOR CASUALTIES.

It was arranged by the Empress of Germany that the Red Cross Society should erect a temporary field hospital at Holtenau in preparation for casualties during the fêtes which took place at the opening of the New Ship Canal. Her Imperial Majesty desired that two doctors, a staff of nurses, and stretcher bearers, should be engaged, accommodation being provided for about forty patients.

### A GOOD REPORT.

The annual report of the Prince Alfred Hospital, Sydney, of which her Majesty the Queen and their Royal Highnesses the Prince and Princess of Wales and the Duke and Duchess of Edinburgh are patrons, contains a gratifying tribute to the matron. Miss McGahey is said to have "devoted herself with much ability, energy, and self-sacrifice to the numerous duties of her department, to the promotion of efficiency in the nursing staff, and to the material and social well-being of the nurses." Courses of evening lectures have been given by doctors on anatomy, physiology, surgical and medical nursing, nursing of sick children, materia medica, and the nursing of insane, nervous, and gynæcological cases. Lectures on nursing are given by the matron, and on invalid cookery by Miss Whiteside. Systematic and practical help has been given to the hospital during the year by numerous lady visitors. They not only distribute flowers to the patients and keep the ward libraries in order, also distributing the books, but some of them assist in the sewing, and repair, as well as make, bed-linen and clothing.

### A MODEL EMERGENCY HOSPITAL.

The managers of the New-York Hospital have recently opened a little Emergency Hospital. Those acquainted with the admirable structure and excellent administration of the parent institution will comprehend that the new branch is likely to prove in every way worthy of it. No pains have been spared in perfecting the arrangements in each department, and this small hospital may be safely regarded as in all respects a model one.

### A NURSES' GUILD.

The Boston branch of the St. Barnabas Guild of Trained Nurses numbers one hundred and seventy-two members. The Guild has been in existence for some nine years, and in the United States alone, eleven hundred nurses have joined it. The association aims at being unsectarian, its objects being summarised as follows: "The aim of the guild is to assist nurses who have received or are in process of receiving the best hospital training, in the cultivation and development of their moral, spiritual, and social powers, without which technical skill is of small avail."

### SHORT ITEMS.

At the Church of the Good Shepherd, Hampstead, flowers, toys, and plants were contributed at the children's flower festal service on a recent Sunday; the offerings were afterwards distributed to local hospitals.—*Nursing Notes* for July contains some interesting correspondence on "District Nurses and Cycles," &c.—The next examination of the London Obstetrical Society will take place on July 10th at 20, Hanover Square.—Miss Pye, who has been Lady Superintendent for twenty-one years of the Ipswich Nurses' Home, has recently resigned the position, in which she has won hearty esteem from her co-workers, and deep gratitude from the sick poor for whom she has done so much.

### Elementary Anatomy and Surgery for Nurses.

By W. McADAM ECCLES, M.B., M.S., F.R.C.S., Lecturer to Nurses, West London Hospital, &c.

### XXIV.—THE NERVOUS SYSTEM.

In order that sensations from the various tissues may be recognised by the owner of those tissues, it is necessary that there be a centre to receive them, and lines along which they may be transmitted; and it is equally true that, to produce a movement of any part of the body, a centre is required to originate or reflect a stimulus and send it forth along a line to the muscle, which must be excited to contract. These functions are carried out by specialised tissue which constitutes the nervous system. In addition to the above, there is in man, and the higher animals, a special part of the nervous system which controls the functions of the blood-vessels, and the various viscera of the thorax and abdomen. The nervous system, therefore, consists of two different but connected parts, the cerebro-spinal and the sympathetic systems. The *cerebro-spinal* portion includes a centre composed of two parts, the brain and the spinal cord, and in addition the nervous lines or cords along which impulses are transmitted, these being known as the cerebral and spinal nerves. The *sympathetic* part, sometimes called the ganglionic system, consists of a double chain of ganglia or groups of nerve cells connected together by intervening nervous cords, and joined by or

FIG. 33.—THE HUMAN BRAIN.
a, Cerebrum; b, Cerbellum; c, Medulla oblongata; d, Spinal Cord.

giving off branches to the spinal nerves in close proximity. From each of the ganglia, moreover, are derived certain nerve trunks which have a great tendency to join together to form a network called a plexus, the terminations of which are finally distributed to the viscera, &c.

The *brain* is the great nerve centre of the body. It is very highly developed in the human subject, and in the adult weighs on the average three pounds. It is contained within the cranium, and is therefore well protected. If the bony wall be removed, a tough, dense membrane, in parts closely adherent to the bone, will be seen. This is called the dura mater. Within this is another very delicate and transparent covering known as the arachnoid membrane, and closely adherent to the brain substance itself is a third membrane, thin, and containing a large number of blood-vessels, by which the cerebral substance is nourished. This is named the pia mater. Between the arachnoid and the pia mater is a space which contains a certain amount of liquid termed the cerebro-spinal fluid, for it is also found in connection with the spinal cord.

The brain itself is a somewhat complicated organ, the chief parts of which are: (1) The cerebrum; (2) the cerebellum, or the lesser brain, consisting of two halves, and lying beneath the posterior part of the cerebrum; (3) the pons Varolii, which in part connects the two portions of the cerebellum; (4) the medulla oblongata, which connects the brain with the spinal cord. (See fig. 33.)

The *cerebrum*, containing the higher motor and sensory centres, and apparently being the seat of the will and the emotions, is composed of two symmetrical halves called hemispheres, right and left, and separated from each other by a very deep cleft or fissure placed vertically, and from the cerebellum behind and below by another fissure placed horizontally. The two hemispheres are connected together. The surface of each hemisphere is very irregular, being thrown into a number of folds or convolutions, whereby its extent is greatly increased; between these folds are clefts, called sulci, into which the pia mater carrying blood-vessels dips. If a section be made through a convolution of a fresh brain, it will be noted that the external layer is greyish in colour, and is, therefore, spoken of as the grey matter, while beneath it the cerebral substance is whitish, forming the white matter. Each half of the cerebrum has, for convenience sake, been divided into lobes which, as they correspond somewhat in area with the bone above them, have been called the frontal, parietal, occipital, and temporo-sphenoidal. The interior of the cerebrum presents certain spaces termed ventricles, which are also filled with cerebro-spinal fluid.

The *medulla oblongata* is a very important part of the central nervous system for in it exist centres which regulate the action of the heart and the respiratory organs, besides having tracts of nerve fibres, whereby the brain is connected with the spinal cord, passing through it.

### Notes and Queries.

The contents of the Editor's Letter-box have now reached such unwieldy proportions that it has become necessary to establish a hard and fast rule regarding Answers to Correspondents. In future, all questions requiring replies will continue to be answered in this column without any fee. If an answer is required by letter, a fee of half-a-crown must be enclosed with the note containing the enquiry. We are always pleased to help our numerous correspondents to the fullest extent, and we can trust them to sympathise in the overwhelming amount of writing which makes the new rules a necessity. Every communication must be accompanied by the writer's name and address, otherwise it will receive no attention.

### Queries.

(180) *Dispensing.*—How can I learn elementary dispensing? Would a book teach me?—*K. L.*

(181) *Medio-Psychological Certificate.*—Will you kindly inform me how I can become a candidate for a certificate from the Medico-Psychological Association?—*Male Attendant.*

(182) *Schoolmistress.*—I am a fully certificated schoolmistress, but am hardly strong enough to go on teaching. Should I be eligible for a post as secretary to a doctor, or assistant secretary to a hospital? I have no science certificate, but have studied anatomy and physiology.—*Edna.*

(183) *Navy.*—How can I get admitted as a nursing sister at Netley?—*C. C.*

(184) *Terms.*—Please tell me of a book which explains medical and surgical terms.—*Nurse A. L.*

(185) *Addresses.*—Kindly tell me addresses of lady superintendents of nursing homes at Cannes and Nice.—*M. S.*

### Answers.

(180) *Dispensing* (*K. L.*).—No. We cannot advise anyone to rest content with "a little" knowledge of dispensing. If you have to do it at all you should be properly instructed. Full information will be found in THE HOSPITAL Nursing Supplement, p. clxxxii., September 24th, 1892; also in "Burdett's Hospital Annual" of last year.

(181) *Medico-Psychological Certificate* (*Male Attendant*).—You had better write to the Hon. Secretary of the Association, Dr. Spence, Burntwood Asylum, Lichfield.

(182) *Schoolmistress* (*Edna*).—You would require to know shorthand and to be a good typewriter to fill either post efficiently, and without this knowledge you would get poorly paid.

(183) *Navy* (*C.C.*).—Write for form of application to the Director General, Medical Department, Admiralty, London.

(184) *Terms* (*Nurse A. L.*).—"The Nurses' Dictionary," published by the Scientific Press. Hoblyn's "Dictionary of Medical Terms," a larger and more expensive book, can also be obtained through the same publishers, at 428, Strand.

(185) *Addresses* (*M. S.*).—You will find the information you require in "Burdett's Hospital Annual" of last year, p. cccxv.

### Nursing in America.—The Convention of Superintendents and Members of the Association.

### THE COMPARATIVE VALUE OF THEORY AND PRACTICE IN THE TRAINING OF NURSES.

By Miss Brennan, Superintendent Bellevue Training School for Nurses, New York.

Twenty-three years ago, it was said, "that no refined educated woman in this country could go through the severe practical training required to fit her to enter the profession of a trained nurse," whereas to-day, in some of our schools, a faint echo of the cry for higher education of women is heard. We take it as a sign of the times, but hope when taking up the higher, the lower education of women may not be neglected. The young woman who enters a training school—mark! it is not a school for nurses, but a training school for nurses—is supposed to do so for the purpose of becoming at the end of two or three years' training a thoroughly efficient nurse, and an intelligent assistant to the attending physician or surgeon, and the aim of all good schools, is, in every way, to help, assist, and train the pupils to become such.

Now, no woman of education and refinement would spend two years in a large city hospital (and only those who have done so can understand what that means) unless she had some compensation in the form of theoretical teaching and study.

An uneducated woman may become a good nurse but never an intelligent one; she can obey orders conscientiously, and understand thoroughly a sick person's needs, but should an emergency arise, where is she? She works through her feelings, and therefore lacks judgment.

In this progressive age training schools cannot afford to stand still any more than other schools and colleges, and each year the graduates should be more skilled, more cultured, and for this reason more practical.

A nurse can always take better care of a patient if she understands the pathology of the disease her patient is suffering from; when typhoid, under no consideration will she allow him to help himself, neither would she, in pneumonia, turn him on his well side, &c., and I hold that all persons in charge of pupil nurses should strive to give a reason for, and explain why this is done or that is not done in each individual case.

The usual length of training is two years, and in that time how much has to be learned both practically and theoretically, but we must discriminate and not sacrifice one for the other.

I have heard the study of the microscope advocated as necessary for the thorough education of the pupil nurse; I acknowledge it to be a most interesting and instructive one, but it requires a great deal of time and much patience. So, unless the hospital be a small one, and the patients few, the pupil nurse will not have the necessary time to devote to it, and would gain much more useful experience if she spent the half-hour she had to spare in studying the character of the pulse in the different patients in the ward, or finding out just why some nurses can always see at a glance that this patient requires her pillow turned, or the next one her position changed.

These are all simple things, necessary to the comfort and well-being of the patient, wherein the microscope cannot help, no matter how proficient the nurse may be in its use. And should the pupil practise her profession after graduating, she will find that even at a private case she has no time to use it, neither would the attending physician expect her to, any more than he would to diagnose the case or write prescriptions.

In the universities and colleges of the world the intention now is to make the teaching far more practical than heretofore; this is particularly so in medical colleges. We all know that the young physician (who most likely has stood first and taken all the honours of his class) when he enters the hospital as interne is utterly unfitted, in spite of his splendid theoretical knowledge, to put into practice what he can so fluently discuss.

Now with the nurse it is different, and just here the point trained comes in (I take it for granted that all training schools have the same fundamental principles), from the very first day she enters the school, she begins with the practical and takes up the theoretical to enable her to give intelligent care to her patients, and to expand her mind by contact with greater minds, in lectures and books, &c., not in any way to make her pedantic or superficial, but to fit her for immediate usefulness when she is graduated.

Theory in conjunction with practice is what we want, and although it is undeniable that theory has done more to elevate nursing than any amount of clinical practice alone could have done, still we must remember that "too much reading tends to mental confusion."

Practice helps to impress and retain in the memory the knowledge obtained by theory, otherwise forgotten without the practical application.

Any one who has been ill knows that the height of good nursing consists principally in what is done for the patient's comfort, outside of the regular orders. A theoretical nurse performs her duty in a perfunctory manner, and may carry out the doctor's orders to the letter, but the patient recognises there is something lacking, and we know that the skilled touch, the deft handling, the keenness to detect changes and symptoms, the ready tact, the patience, unselfishness, self-reliance, and good judgment can be acquired only by much practice, and a nurse without these attributes, despite her wide theoretical knowledge, will never be a successful one.

Now with our superior intelligence and advantages we must not ignore the necessity of possessing a large amount of good plain common sense to form a basis for the education of our nurses, which will hold the theoretical and practical training in a state of equilibrium. Theory fortifies the practical, practice strengthens and retains the theoretical.

### Appointments.

[It is requested that successful candidates will send a copy of their applications and testimonials, with date of election, to The Editor, The Lodge, Porchester Square, W.]

Royal Berks Hospital, Reading.—Miss Eleanor F. Law has been appointed Matron and Superintendent of Nurses at this hospital. She was trained at the London Hospital, and for nearly three years afterwards held the post of Sister-in-Charge of St. John's Hospital for Skin Diseases, Leicester Square, London. Miss Law was subsequently Matron and Superintendent of Nurses at Mercers' Hospital, Dublin, and holds good testimonials. We wish her every success in her new work.

### Where to Go.

Plaistow Maternity Charity and District Nurses' Home.—Annual meeting, by kind permission of Lord Brassey, K.C.B., will be held at 24, Park Lane, on Monday, July 8th, at three p.m.

A sale of work for the above institutions will take place at Courtfield House, Courtfield Gardens, S.W., on Monday, July 15th, at half-past three. Programmes and tickets can be obtained from Mrs. George Hertslet, Courtfield House, and Mrs. Pritchard, 41, Cleveland Square, W.

### Holidays and Health.

[Readers of THE HOSPITAL in need of information about health resorts at home or abroad, or desirous of aid in forming holiday plans, are invited to send queries to EDITOR, 428, Strand, W.C. (marked "Travel" on outside of envelope), which will be answered under this section.]

### II.—THE SHORES OF THE ZUYDER ZEE.

FEW Continental trips can be made with more ease and economy than that to Holland Leaving London (Liverpool Street) at 8.30 p.m., and travelling viâ Harwich and the Hook of Holland, the traveller reaches Amsterdam at 8.30 the following morning. The sea voyage is about eight and a-half hours. Single fare, first class, £1 17s. 6d.; second class, £1 5s.; but tourist tickets, available for sixty days, are issued by the Great Eastern at from £2 9s. 5d. (second return), embracing the principal places of interest in the country. It is not everyone who will have eyes or feeling for the characteristic scenery, but for those who have learned to love the shifting beauty of cloud and sunlight on wide open stretches of country, with the effect of boundless distance, there is a restful contentment even in the quiet monotony of the foreground. A correspondent sends us the following description of the coast north of Amsterdam: "Leaving Amsterdam with the ever busy life of its numerous tree-lined canals, with its multitudinous bridges, its quaint warehouses, and its strangely shaped churches, we journey northwards, intending to get a glimpse of the Zuyder Zee. Crossing the North Sea Canal, and passing through Zaandam with its two or three hundred windmills, we enter the country of the farmers. Everywhere is the flat, flat land, the dead level broken only by the black and white cows, by clumps of willows, by lines of reeds marking the position of ditches and small canals, and here and there by some high-roofed farmhouse, surrounded by trees. Peewits, startled by the passing train, rose with a few gentle flaps of their broad wings, and settled down again; moorhens, gracefully nodding with every stroke of their long-toed feet, were everywhere skimming the marshy pools, and a heron standing knee-deep in a narrow ditch turned its head on its long, stiff neck with an air of wondering curiosity. In about an hour and a half we arrived at Enkhuizen, one of the 'dead cities' of the Zuyder Zee. Leaving the station a round tower with conical windowed roof catches the eye. It commands a gate overlooking the Zuyder Zee, and bears the date 1540, a date that takes us back to times before the great struggle for liberty, and some fifty years before the Spanish fleet, sailing proudly into the Zuyder Zee, was destroyed in front of this very gate. Farther along the sea front the attention is arrested by a large building with quaint old gables, bearing the date 1625. This is a pepper warehouse of the great Dutch East India Company. Amongst the stone decorations of its façade one stands conspicuous. It is a stone tablet with two vessels sculptured upon it—one a small fishing-boat, into which three men are dragging a net; the other, a three-masted ship in full sail. Over it is written, 'Food comes before gain,' pointing to the superiority of home industry over foreign commerce, with a sturdy common sense very characteristic of the old Dutchman. There is abundant evidence in the town that gain as well as food came to the hands of the former dwellers in Enkhuizen. Curious and picturesque homes of wealthy merchants of the past are on every side, many of them with stone-ornamented gables and a tablet bearing the date of erection. These quiet streets of Enkhuizen bear many external signs of the prosperity of the old Dutch merchants; and at Hindeloopen, on the opposite shore of the Zuyder Zee, a very good idea may be formed of their old home life. They were wealthy, but simple. However mush riches might increase the Dutchman made no change in his expenditure. One of the old towers there has been turned into a museum, and in the chief room are figures representing its former occupants. The master is placidly smoking in a tile-lined room, in which at the large open fireplace the daughters are busy preparing his dinner. At the other end of the room heavily-carved cupboards can be seen through the open doors to contain beds, and thus the one apartment sufficed for the cooking, eating, and sleeping of the whole family, one of the wealthiest in the town."

### Queen Victoria Jubilee Institute.

HER MAJESTY has been pleased to approve of the following names being entered on the roll of Queen's Nurses for nursing the sick poor in their own homes:—

ENGLAND.—Superintendent: Georgina M. Shalders, serving Bermondsey; Elizabeth Waller, Birmingham. Nurses: Mary E. Lockington, Lucy A. Bloxam, and Beatrice Handley, Bloomsbury Square; Edith E. Hasted and Emma Kean, Bermondsey; Margaret M. Mounsey, Camberwell; Marian W. Johnston and Charlotte E. Gardiner, Walworth; Jane Coghlan and Isabel Brettell, Hampstead; Anne J. Pinchbeck, Battersea; Alice C. Crowther, Hammersmith; Annie Phelps, Chelsea; Emilie J. Wigmore and Eleanor Herbert, Haggerston; Rebecca M. Emett, Kingston; Mary L. Peet, Woolwich; Sabine A. Badcock, Banbury; Margaret B. Samson, Worthing; Eva Timins, Portsmouth; Florence V. Piton, Aldershot; Ellen M. Ellis, Southampton; Else M R. Böge, Rushden; Dora Woolley, Spalding; Elizabeth Gipps, Bramley; Elizabeth Ross, Handsworth; Gertrude G. Inglis, Bishop Auckland; Mary E. Hooper, Bolton; Jane A. Jones, Mary Rowell, and Dorothy L. Fincken, Leeds; Hilda Johnstone, Bessie L. Broad, and Mary Irvine, Liverpool; Charlotte E. Ashcroft, Edith C. Green, Elizabeth Brayton, Annie F. Broome, Anna Purcell, Mary A. Halsall, Margaret B. Robinson, and Catherine C. White, Manchester.

ENGLAND—RURAL BRANCH.—Nurses: Ellen Oxborrow, serving at Bedworth; Emily J. Grey, Great Bedwyn; Maude M. Piggott, Hasfield; Emily A. H. Forbes, Shenfield; Florence Davey, East Haddon; Florence A. Phillips, Hoo; and Helen M. Kiddle, Chesham.

WALES.—Nurses: Eliza A. Hitchings, serving at Dolgelly; Mary Perkins, Llandilo; Margaret J. Peat, Holyhead; Helen Booth, Ruthin.

SCOTLAND.—Nurses: Elizabeth A, Johnston, Harriette A. Magee, Rachel McKibbin, Mabel D. Low, Margaret D. Little, Cecilia C. Small, Margaret A. Bean, Henrietta Gordon, Jessie A. Wood, Mary B. Tennant, Jemima H. Macnaughton, and Jessie Currie, serving at Edinburgh; Annie D. Hamilton, Margaret D. Hempseed, and Sarah Wright, Glasgow; Katharine McIvor, Tobermory; Marian McDonald, Arisaig; Jane L. Wilson, Lochaber; Jane A. T. Sawyer, Lochwinnoch; Catherine Gordon, Barrhead; Rosina B. Hagart, Kirkcaldy; Joanna S. Anderson, Dumfries.

IRELAND.—Nurses: Alice M. Roe, Susan Murphy, Susan A. Champ, Mary Keane, Julia A Horan, Ellen M. Daly, and Emily F. C. Eastcott, serving in Dublin.

### Paddington Green Children's Hospital.

THE new buildings of the Paddington Green Children's Hospital were formally opened by her Royal Highness the Duchess of Teck on the 1st inst. The erection of the hospital, which will now accommodate forty-eight in-patients, cost £11,000, and the furnishing and other incidental expenses some two or three thousand more. The hospital has, however, been opened entirely free from debt, and purses received by the Duchess of Teck in the course of Monday's ceremony contributed £300 to the general funds. There was a large attendance of visitors on the occasion, and much interest was displayed in the many admirable arrangements by which the skilled nursing of children is facilitated at this well-equipped little hospital.

### The Holloway College.

Last Saturday commemoration was held at the Holloway College for Women, and the prizes were distributed by the Princess Christian. The college, situated on the beautiful wooded slopes of Egham, suggests the ideal of Lilia in Tennyson's "Princess":—

"I would build,
Far off from men, a college, like a man's,
And I would teach them all that men are taught."

Yet this ideal situation and the liberal education has so far failed to render Holloway College a real success, and its spacious buildings are but poorly filled. With the advantages that the college offers in so many respects it is a pity that this should be the case. Lady Frederick Cavendish delivered an admirable address on Saturday, full of common sense and wise counsel, and possibly the kindly sympathy shown by the Princess Christian and other distinguished personages may awaken an interest which will have the best results in spreading the fair fame of the institution.

### Dutch News.

The "White Cross Society" of North Holland, which held its twenty-eighth general meeting in May, under the presidency of Dr. P. J. Barnouw, has added largely to its numbers during the past year. The financial position of the society is not so favourable as it should be, the accounts showing a slight deficiency, due to a falling off in the payments by patients, and in subscriptions from outsiders. The twenty-ninth report of the Society for providing help to those suffering from Diseases of the Eye states that 4,905 patients were attended in the course of the year. The great majority of these patients came from Rotterdam, where the institution is situated, but there were also cases from all parts of Holland, and even from other countries. Another report, hardly so satisfactory, which has just been issued, is that of the "Red Cross Society," which held its annual general meeting a short time ago. It complains of a decline in the subscriptions, as well as in the number of members of the society.

The new matron of the Aldegonde Hospital, Amsterdam, is Mej. M. W. H. Steffhaan, a member of the White Cross Society of Amsterdam.

### Friedenheim.

A fine afternoon enabled those who attended the meeting held on behalf of "Friedenheim" on 29th June to avail themselves of Miss Davidson's invitation to afterwards inspect the beautiful house and grounds which constitute the Home of Peace for the Dying. Situated near Swiss Cottage Station it is comparatively easy of access from all parts of London, and doubtless many who have heard of the work of the Home will respond to the cordial invitation given to them to go again and see for themselves at any time the arrangements for the care and nursing of the sick. Although a sum of £2,000 is required "to place the institution on a sound financial basis," Miss Davidson may be congratulated on having carried on an establishment now containing forty beds, supported by voluntary charity, without getting into debt. The Home is for the reception of hopeless cases of illness, and it provides a peaceful shelter and unvarying kindness for those who, having seen better days, would find a workhouse, however well managed, extremely distasteful.

### Royal British Nurses' Association.

The Secretary of the Royal British Nurses' Association requests us to state that the advertisement of the agenda of the annual meeting on July 24th is unavoidably postponed until *next week*. The attention of all members is drawn to this notice, as in the *Nurses' Journal* for May it was stated that the agenda would appear in the principal medical and nursing papers for the week ending July 6th. As many members are now inquiring whether indoor or outdoor dress is to be worn on the occasion of H.R.H. the President's reception, it may be well to state that *outdoor* dress will be worn, also that members of the Association *only*, and not their friends, can attend the reception, for which invitations to every member have, by H.R.H. the President's directions, been issued.

### An Explanation.

In consequence of the publication of certain statements relating to the conduct of the business of the Royal British Nurses' Association, the executive have just issued a circular to each of the members refuting in detail the accusations brought against the management. Ten pages of foolscap are devoted to explanations and correspondence, and from these it appears that it is obligatory on all members of the General Council to retire in rotation. The suggestion that any exception can be made, even in the case of those ladies who helped to establish the Association, is shown to be impracticable without an alteration in the bye-laws, which can only be made with the consent of the Privy Council after such alterations have been approved by the Association in general meeting.

### The Book World for Women and Nurses.

[We invite Correspondence, Criticism, Enquiries, and Notes on Books likely to interest Women and Nurses. Address, Editor, The Hospital (Nurses' Book World), 428, Strand, W.C.]

The Princess of Wales: A Biographical Sketch. By Mary Spencer Warren. (George Newnes, London.)

Miss Spencer Warren has chosen a most popular theme in her biographical sketch of the Princess of Wales, but, as pointed out in a review on her book in the *Scotsman*, she has no right to claim that it is the "first published volume giving an account of the life of the Princess." Mr. Henry C. Burdett's "Prince, Princess, and People," contains a fuller narrative up to its date of publication than Miss Spencer Warren's work. As Miss Warren herself points out, very little is known of the private life of the Princess of Wales, and therefore the little work in question can hardly claim to be regarded as a biography from this incompleteness. It is, however, pleasant reading as a record of the public life of the beloved Princess, and is likely therefore to find favour. It is well bound and printed, but the portraits of H.R.H. are little short of libellous, and had better have been omitted, seeing that few houses in England do not possess a good photograph of the Princess.

The Art Bible. (George Newnes, Limited.)

We have received the first number of this new periodical issue by Mr. Newnes. It is an illustrated Bible published in twelve parts. The type is exceedingly clear, and is profusely interspersed with drawings after English and foreign artists, illustrating historic incidents, manners and customs, costumes, ceremonies, objects of interest, ancient monuments, and examples in natural history referred to in the Bible. The whole volume will consist of about 1,200 pages, and will be produced at the very small sum of six shillings.

## Figures and Tables

**Fig. 33 f1:**